# The genetic liability to rheumatoid arthritis may decrease hepatocellular carcinoma risk in East Asian population: a Mendelian randomization study

**DOI:** 10.1186/s13075-023-03029-3

**Published:** 2023-03-27

**Authors:** Yuzhuo Zhang, Yudong Zhang, Peng He, Fan Ge, Zhenyu Huo, Guibin Qiao

**Affiliations:** 1 Guangzhou Medical University, Guangzhou, 511436 Guangdong China; 2 Department of Thoracic Surgery & Department of Cardiothoracic Surgery of East Division, The First Affiliated Hospital, Sun Yat-Sen University, Guangzhou, 510062 Guangdong China; 3 National Cancer Center/National Clinical Research Center for Cancer/Cancer Hospital, Chinese Academy of Medical Sciences and Peking Union Medical College, Beijing, 100021 China; 4 Department of Thoracic Surgery, Guangdong Provincial People’s Hospital, Guangdong Academy of Medical Sciences, Guangzhou, 510080 Guangdong China; 5 Shantou University Medical College, Shantou, China; 6 The Second School of Clinical Medicine, Southern Medical University, Guangzhou, 510515 China

**Keywords:** Rheumatoid arthritis, Hepatocellular carcinoma, Cancer risk, Genome-wide association study, Single nucleotide polymorphisms, Mendelian randomization

## Abstract

**Background:**

Patients with rheumatoid arthritis (RA) have a rising possibility of acquiring certain kinds of cancers than the general public. The causal risk association between RA and hepatocellular carcinoma (HCC) remains unknown.

**Methods:**

Genetic summary data from genome-wide association study (GWAS), including RA (*n* = 19,190) and HCC (*n* = 197,611), was analyzed. The inverse-variance weighted (IVW) approach was used as the principal analysis, complemented with weighted median, weighted mode, simple median method, and MR-Egger analyses. The genetic data of RA (*n* = 212,453) was used to verify the results in eastern Asia populations.

**Results:**

The results from the IVW methods indicated that genetically predicted RA was significantly linked with a declined possibility of HCC for East Asians (OR = 0.86; 95% CI: 0.78, 0.95; *p* = 0.003). The weighted median and the weighted mode also supported similar results (all *p* < 0.05). Additionally, neither the funnel plots nor the MR-Egger intercepts revealed any directional pleiotropic effects between RA and HCC. Moreover, the other set of RA data validated the results.

**Conclusion:**

The RA may decrease the risk of being susceptible to the HCC in eastern Asia populations, which was beyond expectation. In the future, additional investigations should be made into potential biomedical mechanisms.

**Supplementary Information:**

The online version contains supplementary material available at 10.1186/s13075-023-03029-3.

## Introduction

Hepatocellular carcinoma (HCC), accounting for about 90% of primary liver cancer, is the fifth most prevalent cancer and the third leading cause of cancer-related mortality globally, with an annual incidence of roughly 500,000 cases [1, 2]. Regarding the global impact of HCC, an initiative to enhance the understanding of HCC risk factors and natural history to establish surveillance projects and manage high-risk populations is ongoing [3]. Meanwhile, rheumatoid arthritis (RA), a chronic, systemic autoimmune disease with global distribution, is one of the leading causes of human disability [4]. In industrialized countries, 0.5 to 1.0% of adults are affected with RA, with an incidence of 5 to 50 per 100,000 annually [5]. Regarding the huge disease burden of HCC and the growing RA epidemic [3, 4], the comorbidities of both diseases may not be uncommon.

Previous studies showed that RA was linked to cancer incidence, and most suggested that it may be correlated with an increased risk of certain kinds of cancers compared to the general population [6–8]. However, it is unknown whether RA is associated with the risk of developing HCC, and few studies paid attention to this issue. A 10-year population-based cohort study investigated the possible causative link between RA and the risk of acquiring HCC, revealing that RA was associated with a lower incidence of HCC (0.66% vs. 1.41% events) [9]. Nonetheless, unmeasurable or undetected confounding factors inevitably exist, which may potentially cause biases. Moreover, featured as chronic inflammation, RA may promote hepatic oncogenesis as there were clues from biochemical studies. Research by Wang et al. indicated that RA patients have significantly increased levels of methyltransferase-like 3 (METTL3) expression, which is positively correlated to disease activity [10], and it has been demonstrated that the METTL3 expression increases in HCC and facilitates cancer cell proliferation, metastasis, and colony formation [11].

Here, we assessed the potential causal association between RA and HCC through two-sample Mendelian randomization (MR). Our study provided new evidence for the relationship between RA and HCC, which may offer novel insight into further mechanistic linking investigations.

## Methods

### Method selection and study design

**Fig. 1:**
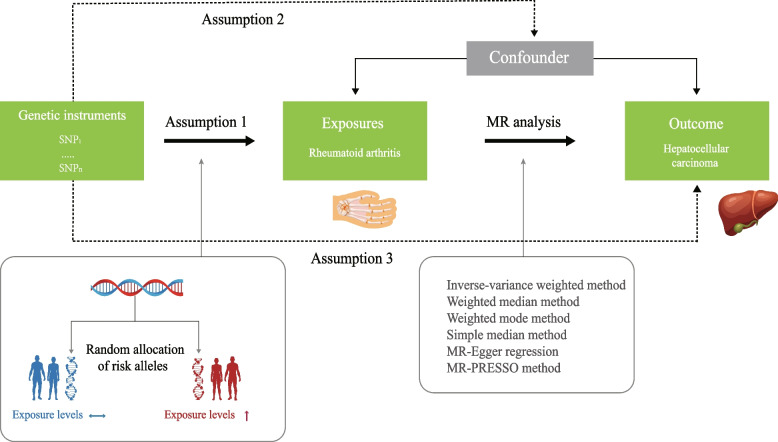
Directed acyclic graph of the MR framework investigating the causal relationship between RA and HCC. Instrumental variable assumptions: (1) the genetic instrument variables (GIVs) must be strongly associated with RA, (2) the GIVs must not be associated with any potential confounder of the RA vs. HCC relationship, and (3) the GIVs should only affect the risk of HCC through RA. SNPs, single nucleotide polymorphisms; RA, rheumatoid arthritis; HCC, hepatocellular carcinoma; MR, Mendelian randomization

The baseline association between RA and HCC is always confounded by unmeasured or irrelevant factors when conducting observational epidemiological studies, which may result in misleading conclusions. Mendelian randomization (MR) provides a new way for causal inference using genetic variants strongly correlated with exposure factors as instrumental variables (IVs) to infer the causal impact of exposure factors on specific outcomes [12]. Because the creation of gametes follows the Mendelian inheritance law of “random allocation of parental alleles to descendant,” genetic variation is unaffected by conventional confounding factors such as environmental influence, socioeconomic factors, and individual behaviors, and its association with outcomes is temporally rational; thus, MR could minimize confounding and reverse causality in traditional observations, providing stronger evidence versus the observational studies [13]. Figure 1 illustrates the research design flow chart.

### Data sources

This study utilized summary-level data from published studies and databases. The informed consent from involved patients was waived based on ethical approval. The genetic data of RA, as well as of HCC, were obtained by meta-analysis of genome-wide association studies (GWAS) including 19,190 samples (3636 cases and 15,554 controls) and 197,611 samples (1,866 cases and 195,745 controls), respectively (accessible at https://gwas.mrcieu.ac.uk/). We extracted the GWAS with the East Asian ancestry group as the population data to diminish potential biases in population stratification. The project details had been described in a previous study published in *Nature* [14]. The summary-level data used to verify the eastern Asia results was taken from the IEU Open GWAS database (https://gwas.mrcieu.ac.uk/), namely rheumatoid arthritis (GWAS ID: bbj-a-151). In addition, we used 11 sets of data to assess the causal relationship between RA and HCC in European populations. Detailed information on GWAS summary data for the European population was provided in Table S3.

### Genetic instrumental variable selection

Single nucleotide polymorphisms (SNPs), which served as IVs for RA, were attained from the Biobank Japan. RA-related SNPs were selected as IVs at the genome-wide significant threshold (*p* < 5 × 10^−8^, indicating a substantial relevance with SNPs and RA) and after screening criteria (the linkage disequilibrium *R*^2^ < 0.001, the length between adjacent SNPs < 10,000 kb). In the summary statistics, HCC-related IVs were removed. After data harmonization, palindromic IVs were excluded due to palindromic with intermediate allele frequencies. Moreover, the *F*-statistic of more than 10 was used as the threshold for excluding genetic variations as potential IVs [15, 16].

### Statistical analyses

MR analyses were conducted with the “MR-PRESSO” [17] and “TwoSampleMR” [18] packages in R version 4.2.1. Principally, the inverse-variance weighted (IVW) technique was adopted to assess the influence of exposure (RA) on the outcome (HCC). Four supplemental analyses were utilized to confirm the findings, which contained weighted mode, weighted median, simple median, and MR-Egger regression. The weighted median method could give relatively robust causal estimations [19]. The MR-Egger regression was used to evaluate the directional pleiotropy of IVs [20]. Moreover, the directed pleiotropy can be adjusted when calculating a causal estimate using the slope of the MR-Egger regression [20, 21]. The MR pleiotropy residual sum and outlier (MR-PRESSO) test was utilized to distinguish horizontal pleiotropy and revised it with outlier removal [18], and the modified Cochran’s *Q* test was adopted for assessing heterogeneity (*p* < 0.05) among SNPs. Finally, we carried out the leave-one-out test to determine if our evaluation was generated from particular SNPs with a substantial impact. The odds ratio (OR) and its 95% confidence intervals (CI) measured the relationships between RA and HCC. *p*-value < 0.05 was considered statistically significant. Additionally, we drew scatter plots to clearly visualize the SNP-related RA and HCC risk.

## Results

### Selection of instruments for RA-related SNPs

Forty-seven RA-related SNPs were extracted as IVs (*R*^2^ < 0.001, *p* < 5 × 10^−8^) from the GWAS study (Table S1). Two SNPs were excluded due to being unavailable in the corresponding data for HCC. Three SNPs (rs13330176, rs1858037, and rs909685) were eliminated from the relevant MR studies after data harmonization due to palindromic with intermediate allele frequencies. The other SNPs’ *F*-statistics were then computed and were all above 10, with values ranging from 49.97 to 3044.57. This showed that the remaining IVs would not likely be subjected to instrument bias and adhered to the first assumption sufficiently [16, 22]. The specifics of the chosen IVs were detailed in Table S1. Ultimately, forty-two SNPs were selected as genetic instruments for MR analyses. The information on RA-related genetic variants and their consequences on HCC was shown in Table 1.

**Table 1:** Characteristic of the genetic variants associated with RA and their effects on HCC (42 SNPs)

SNP	Chr	Position	Effect allele	SNPs-RA			SNPs-HCC		
				***β***	**SE**	***p*-value**	***β***	**SE**	***p*-value**
rs10175798	2	30,449,594	A	0.08	0.01	5.47E − 09	0.07	0.04	0.06
rs11217044	11	118,696,022	C	− 0.13	0.02	3.57E − 15	0.03	0.04	0.53
rs11574914	9	34,710,338	A	0.11	0.02	2.06E − 13	− 0.14	0.08	0.08
rs11889341	2	191,943,742	T	0.13	0.02	6.51E − 19	− 0.09	0.04	0.01
rs1230656	1	114,222,516	A	− 0.20	0.01	3.58E − 41	− 0.03	0.03	0.44
rs13142500	4	10,727,357	C	0.10	0.02	4.92E − 09	0.01	0.03	0.68
rs1571878	6	167,540,842	T	− 0.15	0.01	6.32E − 30	− 0.01	0.03	0.69
rs168962	14	69,282,711	G	− 0.09	0.02	1.69E − 08	0.05	0.03	0.19
rs187786174	1	2,523,811	A	− 0.12	0.02	3.28E − 14	− 0.02	0.03	0.49
rs1893592	21	43,855,067	C	− 0.11	0.02	3.77E − 12	0.05	0.04	0.19
rs1953126	9	123,640,500	C	− 0.08	0.01	9.93E − 10	0.09	0.04	0.01
rs2105325	1	173,349,725	C	0.11	0.02	3.12E − 10	− 0.06	0.06	0.28
rs212389	6	159,489,791	A	0.10	0.02	3.30E − 10	− 0.04	0.07	0.57
rs2228145	1	154,426,970	C	− 0.08	0.01	3.53E − 09	0.01	0.03	0.88
rs2233424	6	44,233,921	T	0.23	0.03	7.69E − 19	− 0.01	0.04	0.83
rs2301888	1	17,672,730	A	− 0.13	0.01	2.29E − 18	− 0.03	0.03	0.30
rs2304256	19	10,475,652	A	− 0.09	0.02	1.27E − 08	0.07	0.03	0.03
rs2317230	1	157,674,997	T	0.08	0.01	2.06E − 08	− 0.02	0.03	0.50
rs2561477	5	102,608,924	A	− 0.09	0.01	1.90E − 09	− 0.01	0.04	0.85
rs2736337	8	11,341,880	C	0.10	0.02	4.79E − 12	− 0.07	0.04	0.08
rs28411352	1	38,278,579	T	0.11	0.02	3.52E − 12	0.03	0.04	0.47
rs3087243	2	204,738,919	A	− 0.13	0.01	1.66E − 22	0.01	0.04	0.88
rs3778753	7	128,580,042	G	0.11	0.01	1.13E − 14	0.01	0.04	0.76
rs3784099	14	68,749,927	A	− 0.10	0.02	7.17E − 10	0.04	0.05	0.48
rs3806624	3	27,764,623	G	0.08	0.01	1.90E − 08	− 0.11	0.05	0.02
rs4239702	20	44,749,251	C	0.11	0.01	9.23E − 15	0.03	0.03	0.36
rs4409785	11	95,311,422	C	0.10	0.02	2.96E − 08	0.08	0.06	0.20
rs5019428	3	17,046,866	A	0.08	0.01	7.27E − 10	− 0.04	0.03	0.30
rs59716545	17	38,031,857	G	0.10	0.01	1.21E − 12	0.01	0.04	0.81
rs61432431	11	128,322,622	C	0.10	0.02	3.62E − 08	− 0.04	0.03	0.22
rs6712515	2	100,806,514	C	− 0.10	0.01	6.96E − 15	− 0.01	0.03	0.76
rs6930468	6	426,268	G	0.09	0.01	5.46E − 11	− 0.06	0.03	0.07
rs706778	10	6,098,949	T	0.09	0.01	1.50E − 10	− 0.01	0.03	0.85
rs71508903	10	63,779,871	T	0.15	0.02	2.45E − 20	− 0.03	0.04	0.42
rs773125	12	56,394,954	G	− 0.09	0.01	4.32E − 10	− 0.04	0.04	0.31
rs7752903	6	138,227,364	G	0.32	0.03	2.70E − 26	− 0.03	0.06	0.65
rs8026898	15	69,991,417	A	0.15	0.02	6.48E − 19	0.07	0.08	0.39
rs8032939	15	38,834,033	C	0.12	0.01	4.86E − 16	− 0.04	0.03	0.29
rs8083786	18	12,881,361	G	0.13	0.02	1.01E − 15	− 0.01	0.04	0.84
rs9267989	6	32,219,320	T	0.70	0.02	0	− 0.18	0.06	0.00
rs947474	10	6,390,450	A	0.10	0.02	1.48E − 08	0.01	0.05	0.87
rs9603616	13	40,368,069	T	− 0.10	0.01	4.56E − 12	0.03	0.04	0.45

*SNP* single nucleotide polymorphism, *SE* standard error

### The effect of RA on HCC

**Fig. 2:**
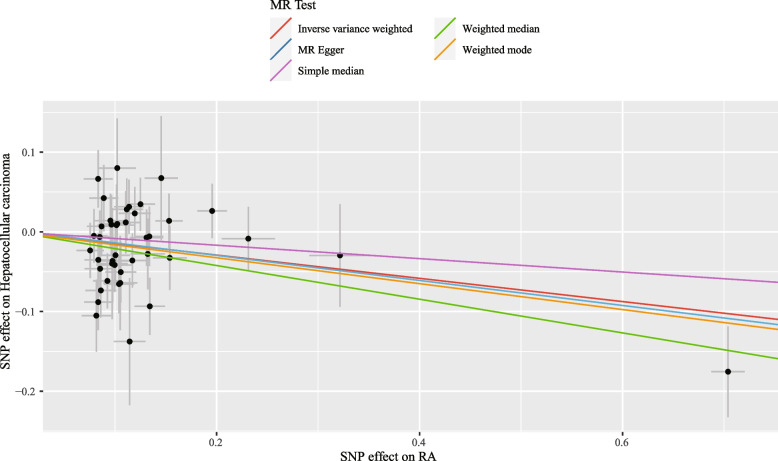
Scatter plot showing the causal effect of RA on hepatocellular carcinoma. SNP, single nucleotide polymorphism; MR, Mendelian randomization

The causal effect of genetic liability to RA on acquiring HCC was obvious from the IVW analysis (OR = 0.86; 95% CI: 0.78, 0.95; *p* = 0.003), showing the presence of RA may be a protective factor for HCC incidence in East Asians (Table 2), and it was visualized in the scatter plot that HCC risk in RA patients decreased (Fig. 2). Moreover, the results of the weighted median method (OR = 0.81; 95% CI: 0.71, 0.92; *p* = 0.002) and the weighted mode method (OR = 0.85; 95% CI: 0.74, 0.98; *p* = 0.032) supported the similar results (Table 2). The MR-Egger method’s intercept *p*-value was 0.89 (> 0.05), implying that IVs did not exist in horizontal pleiotropy. Through the MR-PRESSO method, outliers for HCC were not found. The other RA dataset of East Asian people came to similar results (Table S2), indicating our outcome was steady. Additionally, only one set of data showed a slight increase between RA and HCC risk in European populations, which might be caused by errors. The other data showed that both were uncorrelated. Detailed information is shown in Table S4.

**Table 2:** Effect estimates of the associations between RA and hepatocellular carcinoma

Method	SNPs (*N*)	OR	95%CI	MR *p*-value	Heterogeneity O/*p*-value	Pleiotropy intercept *p*-value
IVW	42	0.864	0.785 ~ 0.952	0.003	53.22/0.095	
Weighted median	42	0.809	0.709 ~ 0.923	0.002		
Weighted mode	42	0.850	0.736 ~ 0.982	0.032		
Simple median	42	0.919	0.792 ~ 1.066	0.267		
MR-Egger	42	0.855	0.708 ~ 1.031	0.109		0.892^b^
MR-PRESSO	42	/	/	0.049^a^		0.099

*SNP* single nucleotide polymorphism, *OR* odds ratio, *CI* confidence interval, *IVW* inverse-variance weighted, *MR* Mendelian randomization, *MR-PRESSO* MR pleiotropy residual sum and outlier

^a^*p*-value of the intercept from MR-Egger regression analysis

^b^*p*-value of MR-PRESSO global test

### Sensitivity analysis

**Fig. 3:**
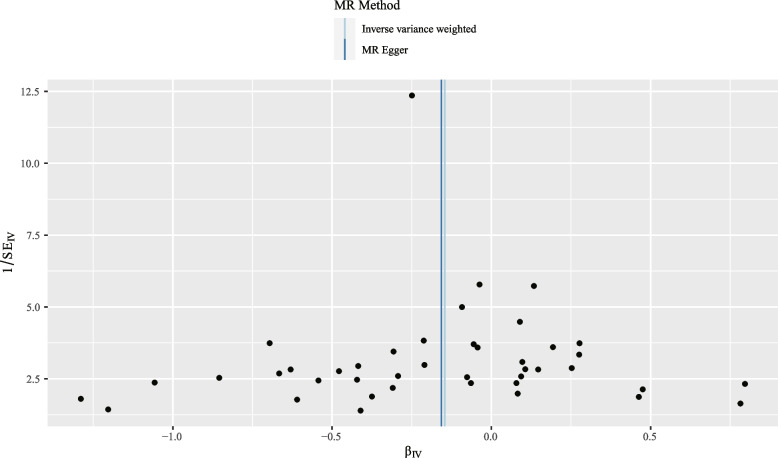
Forest plot of the causal effect of RA-associated SNPs on hepatocellular carcinoma

**Fig. 4:**
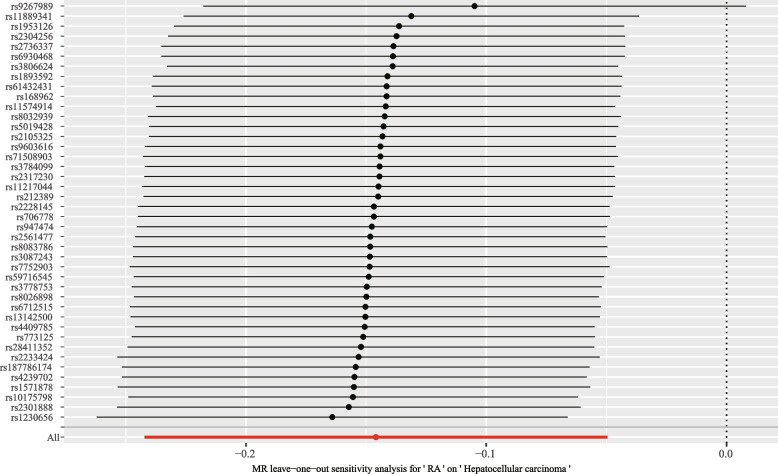
Funnel plot showing no significant heterogeneity among the SNPs. SE, standard error

For the stability of the results, Cochran’s *Q* test demonstrated that no evident heterogeneity was found under the impact of SNPs (*Q* = 53.22; *p* = 0.096) (Fig. 3). Meanwhile, the leave-one-out sensitivity tests identified the influence of every SNP on the overall causal estimates. When deleting a single SNP, no evident shifts in the assessed causal effects were identified (Fig. 4).

## Discussion

As far as we know, this is the first two-sample MR study that comprehensively evaluated the causal association of the genetic predisposition to RA on the risk of developing HCC. Through the 42 SNPs as IVs, we drew the conclusion that genetically predicted RA was significantly linked with a declined possibility of HCC in the population of East Asians, which paves the way for the necessity to conduct relevant molecular mechanisms, access clinical influence, enhancing epidemiological surveillance, and making a public health decision.

Most previous studies showed that RA was associated with an ascending risk of several cancers [23, 24], including lung cancer [25], lymphoma [8], prostate cancer [26], and ovarian malignancy [27], while a few suggested that RA was related to no increase or lower risk of certain cancer [28, 29] like breast cancer and colorectal cancer [6, 30]. As for HCC, only one study we could find was a 10-year population-based cohort study, which showed that RA was associated with a lower risk of HCC (0.66% vs. 1.41% events) [31]. However, there were several shortcomings in the previous research. Owing to the limitations of observational research designs, reverse causation may introduce bias into the findings. Moreover, current epidemiological research has not assessed the impact of potential confounding variables, such as the length of research periods, differences in cancer screening programs between nations, environmental exposures, and data-collecting methodologies, which may distort the underlying link between RA and HCC. Thus, it may be insufficient to draw a sound conclusion.

MR is a novel approach that employs genetic variations of exposures as instruments to identify the effect of certain outcomes. Firstly, the impact of genetics is rather steady and basically immune from the environment influence. The genetic variants are determined when conceived, which is before the disease develops. In addition, MR applies tight quality control conditions and analytic methods and examines the causal effects using a variety of models. Consequently, MR is able to overcome the limits of conventional observational research hampered by reverse causality and confusion as well as generate reliable research findings [12, 32, 33], and this MR study found potential evidence of reduced odds of HCC in RA patients, which might offer insight into successive planned high-quality, large-scale, and long-term cohort studies for investigating the latent relationship between RA and HCC.

Our finding seems to be contrary to the common knowledge that RA leads to a higher risk of cancer. However, the pathogenesis of HCC is likely multifactorial in RA patients. Firstly, the potential protective impact may be by lowering the pathway of liver precancerous diseases, like cirrhosis. The large cohort study [31] indicated that people suffering from RA had a decreased percentage of liver cirrhosis. In addition, using immunomodulatory agents for RA not only affects the human body’s immune status but will also alter the risk of malignancies [8]. Most population-based cohort researches indicated that antirheumatic treatment may not exactly be associated with the cancer recurrence risk in RA patients, either in those receiving conventional synthetic disease-modifying antirheumatic drugs (csDMARDs) or biologic DMARDs (bDMARDs) [31, 34–38] like tumor necrosis factor (TNF) [39–42], and a few studies found that using bDMARDs reduced the overall risk of acquiring malignancies except for hematologic malignancies [43]. Thirdly, it might be attributed to chronic inflammation with immune failure [44], which may motivate tumor suppressor pathways, induce premature and accelerated cell senescence, and ultimately lower the incidence of malignancies in people suffering from RA. These findings implied that biological processes behind the association between RA and HCC were more complicated than immunomodulation and inflammation.

Although the exact mechanism by which RA reduced HCC risk is unclear, a few underlying molecular pathways involving cell growth signal transference may shed light on the relationship. For example, changes in protein levels of G protein-coupled receptor kinase 2 (GRK2), a serine/threonine kinase participating in numerous crucial signaling pathways, may play a crucial role in RA and HCC. On the one hand, the prostaglandin E2-prostaglandin E4 receptor-G protein-coupled receptor kinase 2 (PGE2-EP4-GRK2) signaling pathway has been demonstrated to be relevant in the incidence and progression of RA in animal models. At the functional level, PGE2 binds to the EP4 receptor, triggering excessive GRK2 translocation to the cell membrane and EP4 desensitization, hence downregulating cAMP, malfunctioning FLS, and synovium hyperplasia [45, 46]. On the other hand, Ma et al. discovered that GRK2 inhibits insulin-like growth factor 1 (IGF1)-induced proliferation and migration of HCC cells. Overexpression of GRK2 leads to decreased expression of early growth response-1 (EGR1). Silencing EGR1 mitigates cell proliferation mediated by GRK2 overexpression [47]. In all, the pathogenesis between RA and HCC still needs further investigation.

Additionally, the results should be interpreted with caution due to the following limitations. First, the populations included are of East Asian and European ancestries. Therefore, more studies should be done on other populations. Second, the subgroup analysis is unavailable owing to a lack of clinic-specific data, for example, the lack of gender-specific summary data on instrument exposure related to RA, and the duration of RA exposure, rheumatic activity levels, and therapy impact may have potential bias. More research is needed to confirm a definite causal relationship between RA and HCC.

## Conclusion

Overall, the underlying evidence, a causal effect of reduced probabilities of HCC in RA patients in East Asia, was revealed by this MR research, and it paves the way for the necessity to conduct relevant molecular mechanisms, access clinical influence, enhance epidemiological surveillance, and make a public health decision.

## Supplementary Information

**Additional file 1:** **Table S1.** Selecting instrumental variables related to RA by GWAS threshold (*p*-value < 5 × 10^–8^) (58 SNPs). SNP, single nucleotide polymorphism; Chr, chromosome; EA, effect allele; OA, other allele, SE, standard error. ^a^*R*^2^ were calculated using the following formula: 2 × MAF × (1-MAF) × Beta2, where MAF is the minor allele frequency, Beta is the estimated effect on hip osteoarthritis. ^b^F were calculated using the following formula: R2(N-2)/(1- R2), where R2 is the proportion of variance in hip osteoarthritis explained by each instrument and N is the sample size of the GWAS for the hip osteoarthritis association.

**Additional file 2:** **Table S2.** Verification of the associations between RA and HCC in eastern Asia populations. SNP, single nucleotide polymorphism; OR, odds ratio; CI, confidence interval; IVW, inverse-variance-weighted; MR, Mendelian randomization; MR-PRESSO, MR pleiotropy residual sum and outlier. ^a^*p*-value of the intercept from MR Egger regression analysis. ^b^*p*-value of MR-PRESSO global test.

**Additional file 3:** **Table S3.** Details of studies of RA and HCC for European populations.

**Additional file 4:** **Table S4.** Effect estimates of the associations between RA and HCC in European populations. SNP, single nucleotide polymorphism; OR, odds ratio; CI, confidence interval; IVW, inverse-variance-weighted; MR, Mendelian randomization; MR-PRESSO, MR pleiotropy residual sum and outlier. ^a^*p*-value of the intercept from MR Egger regression analysis. ^b^*p*-value of MR-PRESSO global test.
