# Supplementary material for: The genetic liability to rheumatoid arthritis may decrease hepatocellular carcinoma risk in East Asian population: a Mendelian randomization study
Source: Arthritis Res Ther. 2023 Mar 27;25:49. doi: 10.1186/s13075-023-03029-3 (PMC10041783; doi:10.1186/s13075-023-03029-3)
Supplement: Supplementary file 1 — Additional file 1: Table S1. Selecting instrumental variables related to RA by GWAS threshold (p-value < 5 × 10^–8^) (58 SNPs). SNP, single nucleotide polymorphism; Chr, chromosome; EA, effect allele; OA, other allele, SE, standard error. aR2 were calculated using the following formula: 2 × MAF × (1-MAF) × Beta2, where MAF is the minor allele frequency, Beta is the estimated effect on hip osteoarthritis. bF were calculated using the following formula: R2(N-2)/(1- R2), where R2 is the proportion of variance in hip osteoarthritis explained by each instrument and N is the sample size of the GWAS for the hip osteoarthritis association. [file 13075_2023_3029_MOESM1_ESM.docx]

**Table S1** Seleciting instrumental variables related to RA by GWAS threshold (p-value<5 × 10^-8^) (58 SNPs)

| **SNP** | **Position** | **Chr** | **EA** | **OA** | **EAF** | **β** | **SE** | **p-Value** | **R²** | **F** |
| --- | --- | --- | --- | --- | --- | --- | --- | --- | --- | --- |
| rs28411352 | 38278579 | 1 | T | C | 0.24 | 0.11 | 0.02 | 3.58E-12 | 0.005 | 88.78 |
| rs1230656 | 114222516 | 1 | A | C | 0.67 | -0.2 | 0.01 | 3.93E-41 | 0.017 | 328.11 |
| rs2311481 | 17414063 | 1 | T | C | 0.38 | -0.1 | 0.02 | 1.88E-09 | 0.005 | 97.03 |
| rs2301888 | 17672730 | 1 | A | G | 0.42 | -0.13 | 0.01 | 2.18E-18 | 0.008 | 147.32 |
| rs2228145 | 154426970 | 1 | C | A | 0.4 | -0.08 | 0.01 | 3.47E-09 | 0.003 | 58.02 |
| rs2105325 | 173349725 | 1 | C | A | 0.79 | 0.11 | 0.02 | 3.13E-10 | 0.004 | 70.49 |
| rs2317230 | 157674997 | 1 | T | G | 0.42 | 0.08 | 0.01 | 2.07E-08 | 0.003 | 53.46 |
| rs187786174 | 2523811 | 1 | A | G | 0.33 | -0.12 | 0.02 | 3.30E-14 | 0.006 | 122.34 |
| rs11889341 | 191943742 | 2 | T | C | 0.25 | 0.13 | 0.02 | 6.67E-19 | 0.007 | 130.09 |
| rs10175798 | 30449594 | 2 | A | G | 0.54 | 0.08 | 0.01 | 5.45E-09 | 0.003 | 66.58 |
| rs34695944 | 61124850 | 2 | C | T | 0.27 | 0.11 | 0.02 | 2.61E-13 | 0.005 | 94.77 |
| rs1858037 | 65598300 | 2 | A | T | 0.49 | -0.11 | 0.01 | 1.16E-14 | 0.006 | 120.98 |
| rs6712515 | 100806514 | 2 | C | T | 0.53 | -0.1 | 0.01 | 6.75E-15 | 0.005 | 100.34 |
| rs3087243 | 204738919 | 2 | A | G | 0.4 | -0.13 | 0.01 | 1.66E-22 | 0.009 | 165.67 |
| rs5019428 | 17046866 | 3 | A | G | 0.53 | 0.08 | 0.01 | 7.21E-10 | 0.003 | 66.99 |
| rs3806624 | 27764623 | 3 | G | A | 0.57 | 0.08 | 0.01 | 1.93E-08 | 0.003 | 62.88 |
| rs13142500 | 10727357 | 4 | C | T | 0.49 | 0.1 | 0.02 | 5.00E-09 | 0.005 | 87.81 |
| rs13120727 | 26111351 | 4 | A | C | 0.51 | -0.13 | 0.02 | 8.98E-12 | 0.008 | 154.46 |
| rs7731626 | 55444683 | 5 | A | G | 0.29 | -0.19 | 0.02 | 7.33E-24 | 0.015 | 295.12 |
| rs2561477 | 102608924 | 5 | A | G | 0.31 | -0.09 | 0.01 | 1.90E-09 | 0.003 | 61.67 |
| rs614008 | 31840794 | 6 | T | C | 0.64 | 0.25 | 0.01 | 8.20E-67 | 0.028 | 562.65 |
| rs1571878 | 167540842 | 6 | T | C | 0.55 | -0.15 | 0.01 | 6.13E-30 | 0.012 | 226.14 |
| rs9267989 | 32219320 | 6 | T | G | 0.17 | 0.7 | 0.02 | 1.00E-200 | 0.137 | 3044.57 |
| rs3134970 | 32654202 | 6 | C | T | 0.79 | 0.73 | 0.02 | 1.00E-200 | 0.181 | 4236.59 |
| rs1233386 | 29558190 | 6 | T | C | 0.19 | 0.12 | 0.02 | 3.94E-13 | 0.005 | 87.28 |
| rs1042177 | 33037557 | 6 | T | C | 0.14 | -0.38 | 0.02 | 7.33E-74 | 0.036 | 722.9 |
| rs7752903 | 138227364 | 6 | G | T | 0.04 | 0.32 | 0.03 | 2.72E-26 | 0.008 | 161.67 |
| rs212389 | 159489791 | 6 | A | G | 0.71 | 0.1 | 0.02 | 3.32E-10 | 0.004 | 75.34 |
| rs6930468 | 426268 | 6 | G | A | 0.58 | 0.09 | 0.01 | 5.46E-11 | 0.004 | 80.13 |
| rs34710970 | 32768115 | 6 | G | A | 0.04 | 0.87 | 0.03 | 1.00E-200 | 0.057 | 1166.56 |
| rs2233424 | 44233921 | 6 | T | C | 0.12 | 0.23 | 0.03 | 7.58E-19 | 0.011 | 215.48 |
| rs4713305 | 30252836 | 6 | T | A | 0.24 | 0.24 | 0.01 | 3.32E-58 | 0.021 | 404.12 |
| rs3778753 | 128580042 | 7 | G | A | 0.39 | 0.11 | 0.01 | 1.10E-14 | 0.006 | 112.78 |
| rs2736337 | 11341880 | 8 | C | T | 0.37 | 0.1 | 0.02 | 4.81E-12 | 0.005 | 97.09 |
| rs11574914 | 34710338 | 9 | A | G | 0.26 | 0.11 | 0.02 | 2.08E-13 | 0.005 | 96.7 |
| rs1953126 | 123640500 | 9 | C | T | 0.66 | -0.08 | 0.01 | 9.99E-10 | 0.003 | 60.29 |
| rs706778 | 6098949 | 10 | T | C | 0.45 | 0.09 | 0.01 | 1.50E-10 | 0.004 | 68.79 |
| rs71508903 | 63779871 | 10 | T | C | 0.22 | 0.15 | 0.02 | 2.33E-20 | 0.008 | 155.57 |
| rs947474 | 6390450 | 10 | A | G | 0.83 | 0.1 | 0.02 | 1.45E-08 | 0.003 | 55.44 |
| rs11217044 | 118696022 | 11 | C | T | 0.21 | -0.13 | 0.02 | 3.62E-15 | 0.006 | 111.15 |
| rs4936059 | 128502496 | 11 | G | A | 0.4 | 0.09 | 0.01 | 2.00E-10 | 0.004 | 73.5 |
| rs61432431 | 128322622 | 11 | C | T | 0.18 | 0.1 | 0.02 | 3.64E-08 | 0.003 | 56.54 |
| rs4409785 | 95311422 | 11 | C | T | 0.15 | 0.1 | 0.02 | 2.95E-08 | 0.003 | 49.97 |
| rs773125 | 56394954 | 12 | G | A | 0.35 | -0.09 | 0.01 | 4.38E-10 | 0.004 | 69.34 |
| rs9603616 | 40368069 | 13 | T | C | 0.31 | -0.1 | 0.01 | 4.61E-12 | 0.004 | 83.9 |
| rs3784099 | 68749927 | 14 | A | G | 0.24 | -0.1 | 0.02 | 7.14E-10 | 0.003 | 66.55 |
| rs168962 | 69282711 | 14 | G | C | 0.27 | -0.09 | 0.02 | 1.72E-08 | 0.003 | 55.6 |
| rs8032939 | 38834033 | 15 | C | T | 0.35 | 0.12 | 0.01 | 4.82E-16 | 0.006 | 120.92 |
| rs8026898 | 69991417 | 15 | A | G | 0.21 | 0.15 | 0.02 | 6.52E-19 | 0.007 | 136.75 |
| rs35187679 | 69982695 | 15 | G | A | 0.08 | -0.19 | 0.03 | 4.11E-12 | 0.005 | 92.9 |
| rs13330176 | 86019087 | 16 | A | T | 0.3 | 0.11 | 0.02 | 1.55E-11 | 0.005 | 99.71 |
| rs59716545 | 38031857 | 17 | G | T | 0.4 | 0.1 | 0.01 | 1.20E-12 | 0.004 | 86.2 |
| rs8083786 | 12881361 | 18 | G | A | 0.21 | 0.13 | 0.02 | 1.04E-15 | 0.006 | 109.92 |
| rs2304256 | 10475652 | 19 | A | C | 0.31 | -0.09 | 0.02 | 1.29E-08 | 0.003 | 61.09 |
| rs4239702 | 44749251 | 20 | C | T | 0.69 | 0.11 | 0.01 | 9.00E-15 | 0.006 | 106.58 |
| rs8129030 | 36712588 | 21 | A | T | 0.59 | 0.08 | 0.01 | 2.52E-09 | 0.003 | 62.67 |
| rs1893592 | 43855067 | 21 | C | A | 0.27 | -0.11 | 0.02 | 3.74E-12 | 0.004 | 85.76 |
| rs909685 | 39747671 | 22 | A | T | 0.46 | 0.12 | 0.02 | 6.33E-14 | 0.007 | 131.87 |

SNP, single nucleotide polymorphism; Chr, chromosome; EA, effect allele; OA, other allele, SE, standard error.

^a^R^2^ were calculated using the following formula: 2×MAF×(1-MAF)×Beta2, where MAF is the minor allele frequency , Beta is the estimated effect on hip osteoarthritis.

^b^F were calculated using the following formula: R2(N-2)/(1- R2), where R2 is the proportion of variance in hip osteoarthritis explained by each instrument and N is the sample size of the GWAS for the hip osteoarthritis association.
