# Supplementary material for: The genetic liability to rheumatoid arthritis may decrease hepatocellular carcinoma risk in East Asian population: a Mendelian randomization study
Source: Arthritis Res Ther. 2023 Mar 27;25:49. doi: 10.1186/s13075-023-03029-3 (PMC10041783; doi:10.1186/s13075-023-03029-3)
Supplement: Supplementary file 2 — Additional file 2: Table S2. Verification of the associations between RA and HCC in eastern Asia populations. SNP, single nucleotide polymorphism; OR, odds ratio; CI, confidence interval; IVW, inverse-variance-weighted; MR, Mendelian randomization; MR-PRESSO, MR pleiotropy residual sum and outlier. ap-value of the intercept from MR Egger regression analysis. bp-value of MR-PRESSO global test. [file 13075_2023_3029_MOESM2_ESM.docx]

**Table S2.** Verification of the associations between RA and HCC in eastern Asia populations.

| **Method** | **SNPs(N)** | **OR** | **95%CI** | **MR p-Value** | **Heterogeneity O/p-Value** | **Pleiotropy Intercept p-Value** |
| --- | --- | --- | --- | --- | --- | --- |
| IVW | 6 | 0.789 | 0.649~0.958 | 0.017 | 8.877/0.114 |  |
| Weighted median | 6 | 0.801 | 0.671~0.958 | 0.015 |  |  |
| Weighted mode | 6 | 0.803 | 0.666~0.969 | 0.070 |  |  |
| Simple mode | 6 | 0.790 | 0.587~1.064 | 0.182 |  |  |
| MR-Egger | 6 | 1.027 | 0.627~1.681 | 0.920 |  | 0.319^b^ |
| MR-PRESSO | 6 | / | / | 0.062^a^ |  | 0.246 |

SNP, single nucleotide polymorphism; OR, odds ratio; CI, confidence interval; IVW, inverse-variance-weighted; MR, Mendelian randomization; MR-PRESSO, MR pleiotropy residual sum and outlier. ^a^ p-value of the intercept from MR Egger regression analysis. ^b^ p-value of MR-PRESSO global test.
