# Supplementary material for: The genetic liability to rheumatoid arthritis may decrease hepatocellular carcinoma risk in East Asian population: a Mendelian randomization study
Source: Arthritis Res Ther. 2023 Mar 27;25:49. doi: 10.1186/s13075-023-03029-3 (PMC10041783; doi:10.1186/s13075-023-03029-3)
Supplement: Supplementary file 3 — Additional file 3: Table S3. Details of studies of RA and HCC for European populations. [file 13075_2023_3029_MOESM3_ESM.docx]

| **Table S3**. Details of studies of RA and HCC for European populations. | | | | | | | |
| --- | --- | --- | --- | --- | --- | --- | --- |
| **Disease** | **GWAS ID** | **Year** | **First author** | **PMID** | **Sample size** | **n case** | **n control** |
| RA | ebi-a-GCST000679 | 2010 | Stahl EA | 20453842 | 25,708 | 5,539 | 20,169 |
| RA | ieu-a-834 | 2010 | Stahl EA | 20453842 | 25,708 | 5,539 | 20,169 |
| RA | ebi-a-GCST005569 | 2012 | Eyre S | 23143596 | 47,580 | 13,838 | 33,742 |
| RA | ebi-a-GCST002318 | 2013 | Okada Y | 24390342 | 58,284 | 14,361 | 43,923 |
| RA | ieu-a-832 | 2014 | Okada Y | 24390342 | 58,284 | 14,361 | 43,923 |
| RA | ukb-a-105 | 2017 | Neale | NA | 337,159 | 3,730 | 333,429 |
| RA | ukb-b-9125 | 2018 | Ben Elsworth | NA | 462,933 | 5,201 | 457,732 |
| RA | ukb-d-M13_RHEUMA | 2018 | Neale lab | NA | 361,194 | 1,605 | 359,589 |
| RA | bbj-a-73 | 2019 | Ishigaki K | 24390342 | 8,383 | 2,843 | 5,540 |
| RA | finn-b-M13_RHEUMA | 2021 | NA | NA | 153,457 | 6,236 | 147,221 |
| RA | finn-b-M13_RHEUMA_INCLAVO | 2021 | NA | NA | 218,792 | 6,329 | 212,463 |
| HCC | ieu-b-4953 | 2021 | Burrows | NA | 372,184 | 168 | 372,016 |
