# Supplementary material for: The genetic liability to rheumatoid arthritis may decrease hepatocellular carcinoma risk in East Asian population: a Mendelian randomization study
Source: Arthritis Res Ther. 2023 Mar 27;25:49. doi: 10.1186/s13075-023-03029-3 (PMC10041783; doi:10.1186/s13075-023-03029-3)
Supplement: Supplementary file 4 — Additional file 4: Table S4. Effect estimates of the associations between RA and HCC in European populations. SNP, single nucleotide polymorphism; OR, odds ratio; CI, confidence interval; IVW, inverse-variance-weighted; MR, Mendelian randomization; MR-PRESSO, MR pleiotropy residual sum and outlier. ap-value of the intercept from MR Egger regression analysis. bp-value of MR-PRESSO global test. [file 13075_2023_3029_MOESM4_ESM.docx]

| **Table S4**. Effect estimates of the associations between RA and HCC in European populations. | | | | | | | | |
| --- | --- | --- | --- | --- | --- | --- | --- | --- |
| **Exposure GWAS ID** | **Outcome GWAS ID** | **Method** | **SNPs(N)** | **OR** | **95%CI** | **MR p-Value** | **Heterogeneity O/**  **p-Value** | **Pleiotropy Intercept**  **p-Value** |
| ukb-b-9125 | ieu-b-4953 | IVW | 8 | 1.002 | 0.991~1.012 | 0.715 | 7.773/0.353 |  |
|  |  | Weighted median | 8 | 1.001 | 0.989~1.012 | 0.933 |  |  |
|  |  | Weighted mode | 8 | 1.001 | 0.989~1.012 | 0.908 |  |  |
|  |  | Simple median | 8 | 1.003 | 0.987~1.018 | 0.754 |  |  |
|  |  | MR-Egger | 8 | 0.998 | 0.979~1.016 | 0.803 |  | 0.578^b^ |
|  |  | MR-PRESSO | 8 | / | / | 0.726^a^ |  | 0.512 |
| ukb-d-M13_RHEUMA | ieu-b-4953 | IVW | 4 | 0.996 | 0.977~1.016 | 0.708 | 1.209/0.751 |  |
|  |  | Weighted median | 4 | 0.990 | 0.968~1.012 | 0.366 |  |  |
|  |  | Weighted mode | 4 | 0.989 | 0.965~1.024 | 0.468 |  |  |
|  |  | Simple median | 4 | 0.991 | 0.959~1.024 | 0.628 |  |  |
|  |  | MR-Egger | 4 | 0.999 | 0.942~1.059 | 0.966 |  | 0.943^b^ |
|  |  | MR-PRESSO | 4 |  |  | 0.597a |  | 0.684 |
| ukb-a-105 | ieu-b-4953 | IVW | 4 | 1.003 | 0.990~1.015 | 0.640 | 0.254/0.968 |  |
|  |  | Weighted median | 4 | 1.003 | 0.989~1.017 | 0.669 |  |  |
|  |  | Weighted mode | 4 | 1.001 | 0.986~1.016 | 0.888 |  |  |
|  |  | Simple median | 4 | 1.005 | 0.987~1.023 | 0.621 |  |  |
|  |  | MR-Egger | 4 | 1.002 | 0.980~1.023 | 0.892 |  | 0.901^b^ |
|  |  | MR-PRESSO | 4 |  |  | 0.207a |  | 0.927 |
| ebi-a-GCST002318 | ieu-b-4953 | IVW | 44 | 1.000 | 1.000~1.000 | 0.032 | 37.50/0.708 |  |
|  |  | Weighted median | 44 | 1.000 | 0.999~1.000 | 0.213 |  |  |
|  |  | Weighted mode | 44 | 1.000 | 0.999~1.000 | 0.186 |  |  |
|  |  | Simple median | 44 | 1.000 | 0.999~1.000 | 0.379 |  |  |
|  |  | MR-Egger | 44 | 1.000 | 0.999~1.000 | 0.617 |  | 0.427b |
|  |  | MR-PRESSO | 44 |  |  |  |  |  |
| ieu-a-832 | ieu-b-4953 | IVW | 31 | 1.000 | 0.999~1.000 | 0.319 | 28.99/0.518 |  |
|  |  | Weighted median | 31 | 1.000 | 0.999~1.000 | 0.979 |  |  |
|  |  | Weighted mode | 31 | 0.999 | 0.999~1.000 | 0.932 |  |  |
|  |  | Simple median | 31 | 1.000 | 0.999~1.000 | 0.47 |  |  |
|  |  | MR-Egger | 31 | 0.999 | 0.999~1.000 | 0.784 |  | 0.275^b^ |
|  |  | MR-PRESSO | 31 |  |  | 0.318^a^ |  | 0.496 |
| ebi-a-GCST005569 | ieu-b-4953 | IVW | 13 | 1.000 | 0.999~1.000 | 0.399 | 8.998/0.703 |  |
|  |  | Weighted median | 13 | 1.000 | 0.999~1.000 | 0.867 |  |  |
|  |  | Weighted mode | 13 | 1.000 | 0.999~1.000 | 0.823 |  |  |
|  |  | Simple median | 13 | 1.000 | 0.999~1.000 | 0.340 |  |  |
|  |  | MR-Egger | 13 | 0.999 | 0.999~1.000 | 0.860 |  | 0.34^b^ |
|  |  | MR-PRESSO | 13 |  |  |  |  |  |
| ebi-a-GCST000679 | ieu-b-4953 | IVW | 13 | 1.000 | 0.999~1.000 | 0.398 | 8.998/0.703 |  |
|  |  | Weighted median | 13 | 1.000 | 0.999~1.000 | 0.86 |  |  |
|  |  | Weighted mode | 13 | 1.000 | 0.999~1.000 | 0.82 |  |  |
|  |  | Simple median | 13 | 1.000 | 0.999~1.000 | 0.334 |  |  |
|  |  | MR-Egger | 13 | 0.999 | 0.999~1.000 | 0.86 |  | 0.34^b^ |
|  |  | MR-PRESSO | 13 |  |  |  |  |  |
| ieu-a-834 | ieu-b-4953 | IVW | 8 | 1.000 | 0.999~1.000 | 0.491 | 14.25/0.047 |  |
|  |  | Weighted median | 8 | 0.999 | 0.999~1.000 | 0.925 |  |  |
|  |  | Weighted mode | 8 | 0.999 | 0.999~1.000 | 0.825 |  |  |
|  |  | Simple median | 8 | 1.000 | 0.999~1.000 | 0.520 |  |  |
|  |  | MR-Egger | 8 | 0.999 | 0.999~1.000 | 0.256 |  | 0.057^b^ |
|  |  | MR-PRESSO | 8 |  |  | 0.514^a^ |  | 0.211 |
| bbj-a-73 | ieu-b-4953 | IVW | 31 | 1.000 | 1.000~1.000 | 0.011 | 28.84/0.526 |  |
|  |  | Weighted median | 31 | 1.000 | 1.000~1.000 | 0.007 |  |  |
|  |  | Weighted mode | 31 | 1.000 | 0.999~1.000 | 0.081 |  |  |
|  |  | Simple median | 31 | 1.000 | 0.999~1.000 | 0.121 |  |  |
|  |  | MR-Egger | 31 | 1.000 | 0.999~1.000 | 0.084 |  | 0.292^b^ |
|  |  | MR-PRESSO | 31 |  |  |  |  |  |
| finn-b-M13_RHEUMA | ieu-b-4953 | IVW | 9 | 1.000 | 0.999~1.000 | 0.959 | 9.156/0.329 |  |
|  |  | Weighted median | 9 | 0.999 | 0.999~1.000 | 0.962 |  |  |
|  |  | Weighted mode | 9 | 0.999 | 0.999~1.000 | 0.911 |  |  |
|  |  | Simple median | 9 | 1.000 | 0.999~1.000 | 0.396 |  |  |
|  |  | MR-Egger | 9 | 0.999 | 0.999~1.000 | 0.856 |  | 0.777^b^ |
|  |  | MR-PRESSO | 9 |  |  | 0.960^a^ |  | 0.459 |
| finn-b-M13_RHEUMA_INCLAVO | ieu-b-4953 | IVW | 9 | 1.000 | 0.999~1.000 | 0.843 | 14.63/0.066 |  |
|  |  | Weighted median | 9 | 0.999 | 0.999~1.000 | 0.933 |  |  |
|  |  | Weighted mode | 9 | 0.999 | 0.999~1.000 | 0.899 |  |  |
|  |  | Simple median | 9 | 1.000 | 0.999~1.000 | 0.534 |  |  |
|  |  | MR-Egger | 9 | 0.999 | 0.999~1.000 | 0.722 |  | 0.512^b^ |
|  |  | MR-PRESSO | 9 |  |  | 0.848^a^ |  | 0.224 |
| SNP, single nucleotide polymorphism; OR, odds ratio; CI, confidence interval; IVW, inverse-variance-weighted; MR, Mendelian randomization; MR-PRESSO, MR pleiotropy residual sum and outlier. a p-value of the intercept from MR Egger regression analysis. b p-value of MR-PRESSO global test. | | | | | | | | |
